# Elevated alpha diversity in disturbed sites obscures regional decline and homogenization of amphibian taxonomic, functional and phylogenetic diversity

**DOI:** 10.1038/s41598-023-27946-0

**Published:** 2023-01-31

**Authors:** D. Matthias Dehling, J. Maximilian Dehling

**Affiliations:** 1 Swiss Federal Research Institute WSL, 8903 Birmensdorf, Switzerland; 2 Securing Antarctica’s Environmental Future, School of Biological Sciences, Monash University, Victoria, 3800 Australia; 3 Faculty of Environmental Sciences and Natural Resource Management, Norwegian University of Life Sciences (NMBU), 1432 Ås, Norway; 4 Department of Biology, Institute of Integrated Natural Sciences, University of Koblenz, Koblenz, Germany

**Keywords:** Ecology, Biodiversity, Community ecology, Conservation biology, Ecosystem ecology, Tropical ecology, Wetlands ecology, Herpetology

## Abstract

Loss of natural habitat due to land-use change is one of the major threats to biodiversity worldwide. It not only affects the diversity of local species communities (alpha diversity) but can also lead to large-scale homogenization of community composition (reduced beta diversity) and loss of regional diversity (gamma diversity), but these effects are still rarely investigated. We assessed the impact of land-use change on taxonomic, functional and phylogenetic diversity of amphibians in Rwanda, both on the local (community-level) and regional scale (country-wide). Alpha diversity in local communities was higher in farmland than in natural habitats; however, species turnover among farmland sites was much lower than among natural sites, resulting in highly homogenized communities and reduced taxonomic, functional and phylogenetic gamma diversity in farmland across Rwanda. Amphibians found in farmland were mostly disturbance-tolerant species that are widespread in eastern Africa and beyond. In contrast, most of the regionally endemic frog species that make this region a continent-wide hotspot of amphibian diversity were found only in the natural habitats. Ongoing habitat conversion might result in further homogenization of amphibian communities across sub-Saharan Africa and the loss of regional endemism, unique evolutionary lineages, and multifunctionality.

## Introduction

Loss of natural habitat due to anthropogenic land-cover change, such as deforestation and conversion into farmland, is a major driver of species loss^1,2^, with severe impacts on global biodiversity^3,4^. Loss of biodiversity has severe negative impacts on ecosystems functioning which, in turn, poses a threat to human well-being^5–13^. Disturbances such as land-cover change might not only cause species loss in a community (reduced alpha diversity) but can also change its composition due to species replacements^2,14^. Such species replacements can shift the functional composition of a community even if local species richness remains the same^15^. Large-scale land-cover change across communities and habitat types can then result in the replacement of locally restricted habitat specialists by a reduced set of disturbance-tolerant, widespread generalists, resulting in reduced beta diversity and biotic homogenization^16–18^. Moreover, natural communities from different habitats complement each other in their ecosystem functions on the regional scale^19^, and maintaining this “multifunctionality”^18^ at high levels requires a diverse set of communities, with both high alpha and high beta diversity^10,17,20^. Biotic homogenization, in contrast, reduces complementarity among communities from different habitat types, leading to a decrease in multifunctionality^18,21,22^. Although human-driven loss of beta diversity and biotic homogenization of communities could be as widespread and as detrimental as the loss of alpha diversity^2,4,12^, studies on the effect of biotic homogenization on multifunctionality are still widely lacking^19,23–25^.

Assessments of the diversity of ecological assemblages across habitat types—traditionally assessed via species richness—are increasingly extended to include measures of functional and phylogenetic diversity. Functional diversity measures the combination of species traits that are associated with adaptations to the environment and species’ roles in ecological processes^26^, and communities with higher functional alpha diversity provide a wider range of ecological functions^27,28^. Phylogenetic diversity measures the diversity of evolutionary lineages^29–31^, which represents species’ evolutionary history as well as their potential adaptability to environmental changes^32–36^. Since these different facets of diversity can idiosyncratically respond to disturbances^14,37,38^, consideration of taxonomic, functional and phylogenetic diversity might provide complementary insights into the effect of land-use change on species communities, but they are rarely included together in assessments of biotic homogenization.

Amphibians are paramount for wetlands and aquatic habitats because they provide key ecological functions, especially in tropical regions^39,40^. Amphibian diversity worldwide is threatened by climatic change, invasive species and diseases^41–43^, and especially by habitat loss^1,44^. Despite the functional importance of amphibians, the effect of anthropogenic habitat alterations on amphibian communities is poorly understood: at the local scale, species richness in disturbed sites was found to be lower^45–50^, unchanged^51,52^, or even higher^53,54^ compared to natural sites. In addition, all studies so far have only focused on changes in local alpha diversity, without considering changes in the species composition of amphibian communities nor the potential effects of habitat alteration on the homogenization and multifunctionality of amphibian communities.

We investigated the effect of habitat alteration on the diversity and composition of amphibian communities in Rwanda. Rwanda is the most densely-populated country in continental Africa, with currently over 74 percent of the land surface exploited for agriculture, and the remaining natural habitats threatened by growing demand for subsistence agriculture, livestock grazing, and fuel extraction^55^. Using data from 37 sites across the country, we compared the taxonomic (species richness), functional, and phylogenetic diversity of amphibian communities between natural sites (natural forest, savannah) and farmland (agricultural *marais*), considering changes in the local diversity of amphibian communities (alpha diversity), pairwise differences in community composition (beta diversity), and changes on the regional scale across Rwanda (gamma diversity). We found that alpha diversity of amphibians was higher in farmland sites than in natural sites. However, since amphibian communities in farmland sites were highly homogenized, gamma diversity of amphibians across Rwanda was much lower in farmland sites compared to natural sites. We also compiled data on the distribution of amphibians across sub-Saharan and transformed them to a grid with a resolution of a 1°. For each grid cell, we calculated taxonomic and phylogenetic alpha diversity and complementarity (the degree to which the taxonomic and phylogenetic composition of a grid cells overlaps with that of other grid cells).﻿ The natural amphibian community of Rwanda showed one of the highest levels of taxonomic and phylogenetic diversity and complementarity across sub-Saharan Africa.

## Results

### Comparison of alpha and gamma diversity

We recorded a total of 42 amphibian species at the study sites: 39 in natural habitats (22 in forest, 17 in savannah) and 19 in farmland. Fifteen species (eleven savannah and four forest species) were shared between natural sites and farmland; three species (*Hyperolius lateralis*, *Ptychadena porosissima*, *P. uzungwensis*) were exclusively found in farmland.

**Figure 1:**
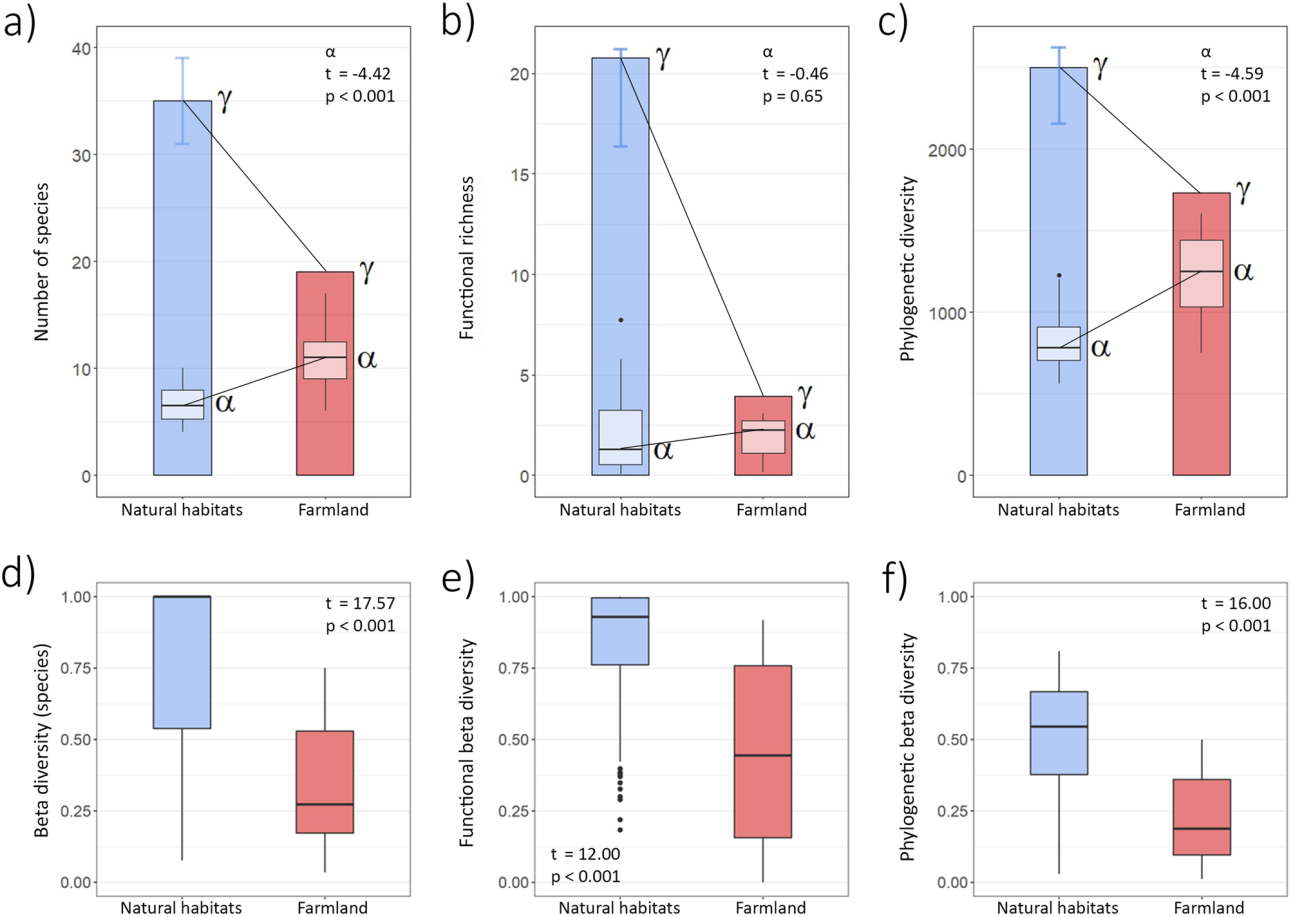
Alpha and gamma diversity (**a**–**c**) and pairwise beta diversity (**d**–**f**) of amphibian communities in natural habitats (natural forest, natural savannah; blue) vs farmland (red) across Rwanda. Diversity is measured as (**a**, **d**) taxonomic diversity (number of species), (**b**, **e**) functional diversity (functional richness), and (**c**, **f**) phylogenetic diversity (Faith’s PD) both on the level of local communities (alpha diversity) and once pooled across Rwanda (gamma diversity). Gamma diversity is shown as colored bars; median and standard deviation of local alpha diversity (n = 15) are shown as lighter boxplots within bars. Gamma diversity in natural habitats represents the median of 1000 sampled combinations of seven and eight forest and savannah sites; error bars indicate observed range. Alpha diversity was consistently higher in farmland than in the natural habitat, whereas gamma diversity was consistently higher in natural sites than in farmland (**a**–**c**). Correspondingly, beta diversity was consistently higher in natural sites than in farmland (**d**–**f**).

Alpha diversity in natural habitats was generally lower than in farmland (*species richness*, natural sites: median (inter-quartile range) 6.5 (2.75), range 4–10, farmland: 11 (3.5), 6–17; two-sided t-test, t = −4.42, *p* < 0.001, Fig. 1a; *functional diversity*, natural sites: 1.26 (2.69), 0.06–7.72, farmland 2.24 (1.63), 0.138–3.06; t = −0.46, *p* = 0.65, Fig. 1b; *phylogenetic diversity*, natural sites: 783 (205), 561–1228, farmland: 1248 (411), 747–1604; t = −4.59, *p* < 0.001, Fig. 1c). In contrast, gamma diversity was higher in natural habitats than in farmland (*species richness*, natural sites: mean ± SD 35 ± 4, farmland: 19, Fig. 1a; *functional diversity*, natural sites: 20.8 ± 0.86, farmland: 3.9, Fig. 1b; *phylogenetic diversity* natural sites: 2501 ± 56, farmland: 1731, Fig. 1c). In accordance with the previous two findings, beta diversity among sites was higher in natural habitats than in farmland (*species richness,* natural sites: median (inter-quartile range)﻿ 1 (0.46), 0.07–1, farmland: 0.27 (0.36), 0.03–0.75; t = 17.57, *p* < 0.001, Fig. 1d; *functional diversity,* natural sites: 0.93 (0.23), 0.18–1.00, farmland: 0.44 (0.60), 0.00–0.92; t = 12.00, *p* < 0.001, Fig. 1e; *phylogenetic diversity,* natural sites: 0.55 (0.29), 0.03–0.81, farmland: 0.19 (0.26), 0.01–0.50; t = 16.00, *p* < 0.001, Fig. 1f).

**Figure 2:**
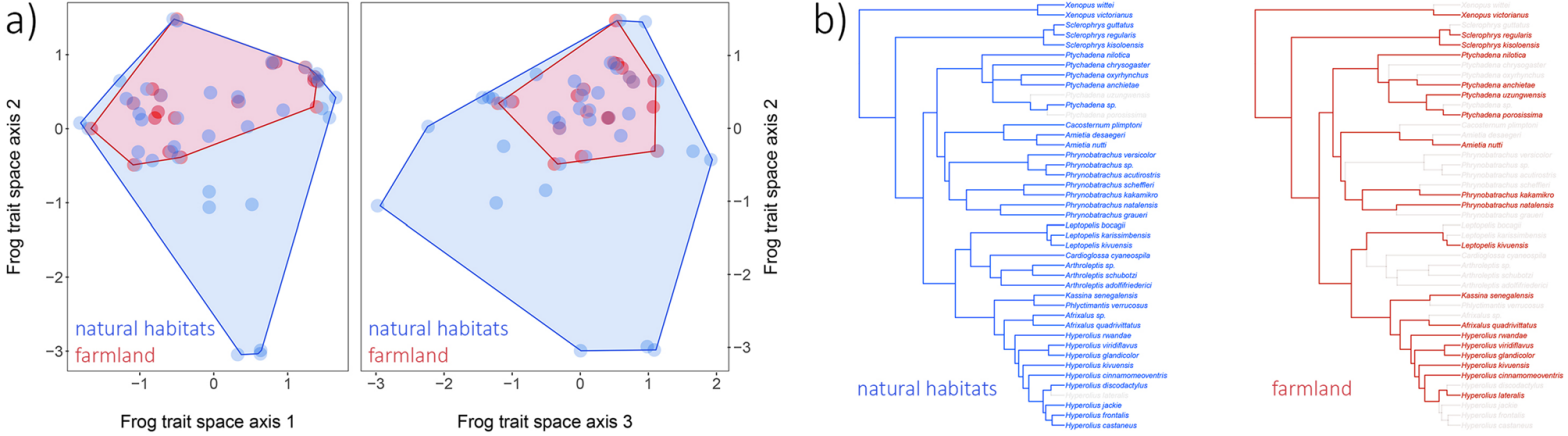
Functional and phylogenetic diversity of amphibian communities in Rwanda. (**a**) Diversity of functional-trait combinations (functional richness) of amphibian species in natural sites (blue) vs. farmland (red) across Rwanda. Species are projected into a three-dimensional trait space where they are arranged according to the similarity in their trait combinations (left: axes 1 and 2, right: axes 2 and 3). The trait combinations of frogs found in farmland are much smaller than, and completely nested within, the trait combinations of species found in the natural sites. (**b**) Phylogenetic tree of the frog species found in natural sites (blue) and farmland (red) across Rwanda. The farmland holds most of the major lineages but the number of branches within the lineages is greatly reduced.

Local species richness was closely correlated with phylogenetic alpha diversity (natural sites, R^2^ = 0.81, *p* < 0.001; farmland, R^2^ = 0.91, *p* < 0.001); functional alpha diversity was positively, but more weakly, correlated with local species richness (natural sites, R^2^ = 0.38, *p* = 0.002; farmland, R^2^ = 0.77, *p* < 0.001) and phylogenetic alpha diversity (natural sites, R^2^ = 0.51, p < 0.001; farmland, R^2^ = 0.90, *p* < 0.001). The functional-trait combinations of amphibians in farmland were completely nested within those from natural sites (Fig. 2a). Most of the major phylogenetic lineages that were present in natural sites were still represented in the farmland, albeit by less than half of the branch lengths (Fig. 2b). Species-accumulation curves showed that our sampling of amphibian communities in farmland sites, but not in natural sites, reached saturation after 15 sites (Supplementary Information 1, Fig. S1). The observed differences between natural sites and farmland are therefore conservative estimates and can be expected to be even more severe.

### Species and phylogenetic complementarity

**Figure 3:**
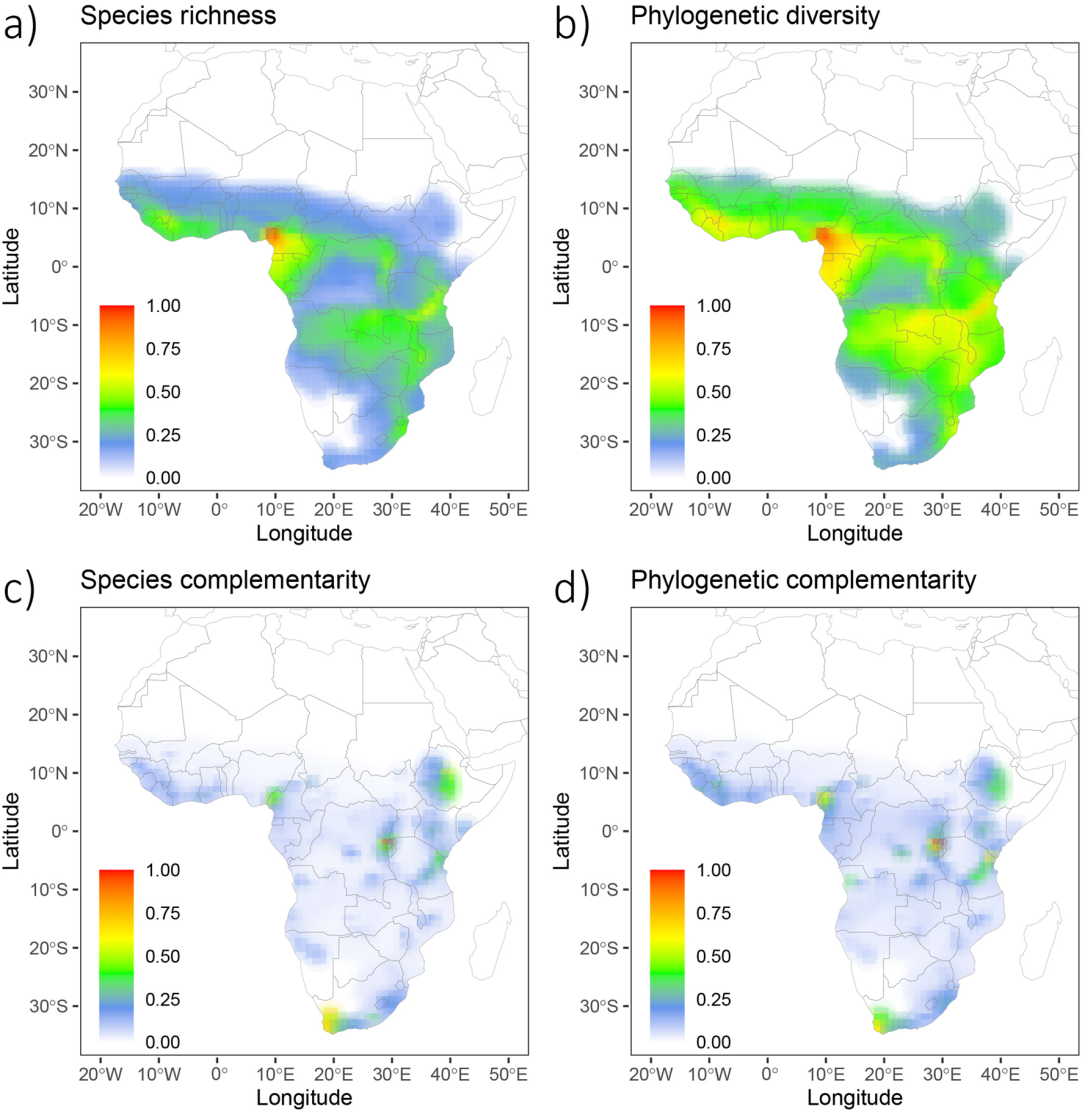
Taxonomic (species richness) and phylogenetic diversity and complementarity of amphibian species across sub-Saharan Africa at 1° resolution. All values are scaled relative to the highest observed value. (**a**) Species richness and (**b**) phylogenetic diversity (Faith’s PD) are measured as the number of frog species whose ranges overlap in a grid cell and their combined branch lengths in the frog phylogeny, respectively. Species richness and phylogenetic diversity peak in western Central Africa. (**c**) Species complementarity and (**d**) phylogenetic complementarity describe the contribution of each grid cell to overall species richness and phylogenetic diversity, respectively, relative to the number of the diversity found in the grid cell. Grid cells that include a high percentage of species/phylogenetic lineages that are shared with few other grid cells show high complementarity; grid cells with a high percentage of species/phylogenetic lineages that occur in many other grid cells show low complementarity. Species and phylogenetic complementarity peak in the Albertine Rift (including Rwanda), East Africa, and the Cape Region. Figure created with R 4.1^78^.

Compared to other areas in sub-Saharan Africa, the amphibian communities in Rwanda showed moderate species richness and phylogenetic diversity (Fig. 3a,b). However, the area along the Albertine Rift (including the natural habitats in Rwanda) showed the highest species and phylogenetic complementarity, i.e. this region had the highest percentage of species and lineages with restricted ranges, whereas large areas in sub-Saharan Africa showed low levels of complementarity (Fig. 3c,d). Species richness and phylogenetic diversity were positively, but weakly, correlated with species (R^2^ = 0.04, *p* < 0.001) and phylogenetic complementarity (R^2^ = 0.07, *p* < 0.001), respectively.

## Discussion

Habitat deterioration due to conversion of natural habitats into farmland drastically reduced the taxonomic, functional, and phylogenetic diversity of amphibians in Rwanda. Amphibian communities in farmland showed higher local alpha diversity than amphibian communities in natural habitats, which could misleadingly suggest that disturbed sites in farmland support diverse frog communities. However, communities in farmland were highly homogenized and showed almost the same species composition across the entire country. As a consequence, the regional taxonomic, functional and phylogenetic gamma diversity of amphibians in farmland across Rwanda was much lower than in natural sites, even though farmland covers a much larger area. The high alpha diversity in the farmland therefore masked the detrimental effects of habitat alterations on amphibian diversity in Rwanda^19,56^. This underlines that considering only local alpha diversity as an indicator for the effects of disturbance can be misleading as it ignores potential changes in species composition at both the local and the regional scale—studies on the effect of habitat alteration on species communities should therefore include measures of beta diversity to fully assess the impact on diversity and multifunctionality at the regional scale^10,18,19,21,22,24^.

The strong decrease in functional diversity from natural forest to farmland shows that species in natural sites fulfil many unique functional roles that cannot be fulfilled by species in farmland^57^. While in some cases disturbed sites might hold species communities with functional roles that are complementary to those in natural habitats^14,15^, this was not the case for amphibians in Rwanda: the functional-trait combinations of frogs from farmland were entirely nested within those from natural sites, indicating that habitat alteration only leads to a loss, not a shift, of functional roles in the amphibian communities—i.e. a complex system replaced by a simplified one^16^. On the country scale, the homogenization of amphibian communities driven by the conversion of natural habitat into farmland hence resulted in a severe loss of amphibian multifunctionality.

The decrease of phylogenetic gamma diversity in farmland was less severe compared to the loss of taxonomic and functional gamma diversity, and it was limited to the loss of branches within the major lineages, indicating that some of the locally extinct species were replaced by closely-related species from the same major lineage. Under the assumption that closely-related species fulfill similar functional roles, this could lead to the false impression that in disturbed sites most ecological functions are still maintained. However, the local extinction of evolutionary lineages driven by habitat conversion in Rwanda included four highly-adapted lineages with restricted geographic ranges. These comprised the only representative of the genus *Cacosternum*, a highly-adapted savannah specialist, as well as regionally endemic forest specialists with adaptations to stream breeding or direct development in the genera *Arthroleptis* and *Cardioglossa*, in the basal lineage of *Phrynobatrachus*^58^, and in the *Hyperolius-castaneus* group^59^. The comparison of functional diversity revealed that these species show trait combinations that are very different from those of other species, indicating that they fulfill functional roles that cannot be replaced by closely-related, disturbance-tolerant species. Moreover, our comparison of alpha diversity showed that phylogenetic alpha diversity and local species richness were only moderately-well correlated with functional alpha diversity. This suggests that using species richness or phylogenetic diversity as a proxy for functional diversity might not fully reflect the impact of disturbance on amphibian communities revealed by the analysis of functional diversity^60^.

The severe loss of regional taxonomic, functional, and phylogenetic gamma diversity is in line with previous studies showing that homogenization reduces regional diversity by replacing unique endemic species with widespread, abundant and often invasive species^16,61–63^. The Rwandan agricultural landscape is a highly homogenous environment, which allows disturbance-tolerant amphibians to disperse without barriers^54^ and expand their ranges^2,16^—in fact, two of the species found in farmland (*Ptychadena porosissima*, *P*. *uzungwensis*) possibly colonized Rwanda only after the extensive modification of natural habitat^53,64^. Amphibian species found in Rwandan farmland show common characteristics of species that thrive in human-altered environments, such as ancestral reproductive modes with high fecundity and the ability to breed throughout the year, and wide climatic niches which facilitate large geographic ranges in eastern Africa and beyond^16,50,61^; none of these species is currently threatened by extinction (IUCN Red List status “Least Concern”^65^). These disturbance-tolerant species in farmland thus represent the “winners”^16^ among the Rwandan amphibians. In contrast, the species restricted to natural habitats show characteristics such as specialized reproduction modes, including small clutches of large eggs, direct development, highly seasonal breeding, and reproduction in streams. These species from natural habitats thus represent the “losers”^16^, and the losers outnumber the winners by a wide margin. Most Rwandan amphibians from natural sites have very small geographic ranges, including several species that are endemic to the mountains of the Albertine Rift^59,64,66,67^. Our comparison of taxonomic and phylogenetic diversity on the continental scale showed that the natural amphibian community of the Albertine Rift constitutes one of the most complementary assemblages across Africa. If the remaining natural habitats of the region continue to be altered, this unique—and to a large part endemic—amphibian fauna will be lost and replaced by a small number of generalist species that are widespread across sub-Saharan Africa.

## Material and methods

### Sampling of amphibian communities

We sampled amphibian communities in natural habitats and farmland across Rwanda. Natural habitats included swamps in natural forest (hereafter “forest”) in three national parks (Volcano NP, Gishwati-Mukura NP, Nyungwe NP; n = 11, 1804–3031 m a.s.l.) and wetlands and ephemeral ponds in natural savannah (hereafter “savannah) in Akagera NP (n = 11, 1287–1642 m a.s.l.). Farmland sites (traditional *marais*) were distributed all over Rwanda and typically consisted of a mosaic of crop patches (< 0.1 ha) separated by small irrigation channels (n = 15, 1292–2348 m a.s.l.). We selected farmland sites from a similar elevational range as the natural sites. We sampled each site six times at different times of the year, both at the beginning of the short rainy season (September, October) and at the height of the long rainy season (March, April). We assessed the presence of species at each site through visual and acoustic encounter surveys^68^. Each sampling was conducted by a single individual for two hours starting 20 min after sunset. Each site was investigated by slowly walking along water bodies or through areas with a lot of calling activity, noting every species encountered. Calling males were identified by their unique advertisement calls. In addition, we made sound recordings every 30 min for five minutes that were later checked for advertisement calls. To take into account seasonality of species activity, most sites—including all sites in Akagera NP, which are most strongly affected by seasonality—were also sampled during the short dry season (December, January) and at the beginning (May) and end (September) of the long dry season. However, since these dry-season samples did not yield any additional species, we excluded them from the analysis.

### Functional traits of frogs

For all frog species encountered, we collected ecological, morphological and life-history traits related to resource use and functional roles of species, following Ernst et al.^51^ and Cadotte et al.^27^: microhabitat use, calling site, breeding seasonality, egg deposition site and type, tadpole type, snout-vent length, head width, hind-limb length, hand and foot webbing, terminal disks (Supplementary Information 1, Table S1). We obtained life-history traits in the field and morphological traits from specimens collected at the sites. For morphometrics, we measured between 1 and 87 (median 8) specimens from each sex of each species. Additional life-history information was taken from Channing & Howell^69^.

### Amphibian phylogeny

For all frog species encountered, we compiled a phylogeny using the consensus tree from Jetz & Pyron^70^. Missing species (n = 3) were added at the tip of the branch of the most closely-related species.

### Comparison of alpha and gamma diversity

For each site, we determined the taxonomic, functional, and phylogenetic alpha diversity of amphibians. We measured taxonomic alpha diversity as species richness, i.e. the total number of frog species found at a site. We measured functional alpha diversity of amphibians as the diversity of their functional-trait combinations. We first calculated the pairwise differences in trait combinations between all frog species using Gower’s distance (since our functional traits included continuous and categorical data^71^). We then used non-metric multidimensional scaling to project all frog species into one common three-dimensional trait space where they were arranged according to the differences in their trait combinations. We calculated the functional-trait diversity for each site as functional richness, i.e. the volume of a convex hull in the three-dimensional trait space that includes all frog species found at that site^71,72^. We measured phylogenetic diversity as Faith’s PD^73^, the combined length of all branches that connect the frog species found at a site.

For each habitat category, we pooled species across all sites to calculate the total species richness (taxonomic gamma diversity), total functional richness (functional gamma diversity), and total phylogenetic diversity (phylogenetic gamma diversity) found across Rwanda. For the farmland sites, we used all 15 sites sampled in farmland. Since the combined number of natural sites (22) was higher than the number of farmland sites, which could lead to a higher observed gamma diversity, we used subsamples of 15 natural sites (1000 combinations of 7 and 8 sites from forest and savannah) for the gamma-diversity comparison. To test whether our samples of frog communities were representative of the different habitat types (farmland, natural habitat), we calculated species-accumulation curves (Supplementary Information 1, Fig. S1). Since some of the forest plots were located at higher elevations than the highest farmland sites and since vertebrate communities usually show a turnover in species composition along elevational gradients^74^, we repeated the comparison of alpha and gamma diversity without the forest plots above 2500 m elevation. These comparisons yielded very similar results (Supplementary Information 2). For each habitat category, we calculated pairwise taxonomic, functional, and phylogenetic beta diversity between all sites as Soerensen dissimilarity using the betapart R package^75^. We compared differences in alpha and beta diversity between natural and farmland sites with two-sided t-tests.

### Species and phylogenetic complementarity across sub-Saharan Africa

We used data from the IUCN^65^ to map the distribution of all amphibian species in sub-Saharan Africa (< 20° N) in 1° grid cells. In order to compare the natural and disturbed species communities in Rwanda with the rest of sub-Saharan Africa, we replaced the three grid cells for Rwanda with one grid cell that included the species found in natural habitats and two grid cells that included the species found in farmland (which is a conservative approach, as the farmland occupies a much larger area relative to natural habitat in Rwanda). For each grid cell, we calculated species richness and phylogenetic diversity of amphibians. In addition, we calculated the complementarity in species and phylogenetic diversity: the contribution of each grid cell to total species richness and phylogenetic diversity across Africa, measured relative to the species richness and phylogenetic diversity found in the grid cell^76,77^. Complementarity is high for grid cells that include a high percentage of species or phylogenetic lineages with a small distribution, and it is low for grid cells that mostly include widespread species and lineages. We could not consider the functional diversity of amphibians in this analysis because trait data for all African amphibians were not available.

We used R 4.1^78^ for all analyses.

## Supplementary Information

Supplementary Information.

## Data Availability

Data on amphibian traits and occurrence in Rwanda will be permanently archived in a public repository once the paper is accepted for publication. The phylogeny and distribution maps for African amphibians are available from the sources cited in the manuscript. Code used in the analyses was sourced from existing statistical packages and repositories that are cited in the manuscript.
