# Supplementary material for: Elevated alpha diversity in disturbed sites obscures regional decline and homogenization of amphibian taxonomic, functional and phylogenetic diversity
Source: Sci Rep. 2023 Jan 31;13:1710. doi: 10.1038/s41598-023-27946-0 (PMC9889332; doi:10.1038/s41598-023-27946-0)
Supplement: Supplementary file 1 — Supplementary Information. [file 41598_2023_27946_MOESM1_ESM.pdf]

Title: Elevated alpha diversity in disturbed sites obscures regional decline and homogenization of amphibian taxonomic, functional and phylogenetic diversity

Authors: D. Matthias Dehling, J. Maximilian Dehling

Supplementary Information 1

Supplementary figures and tables

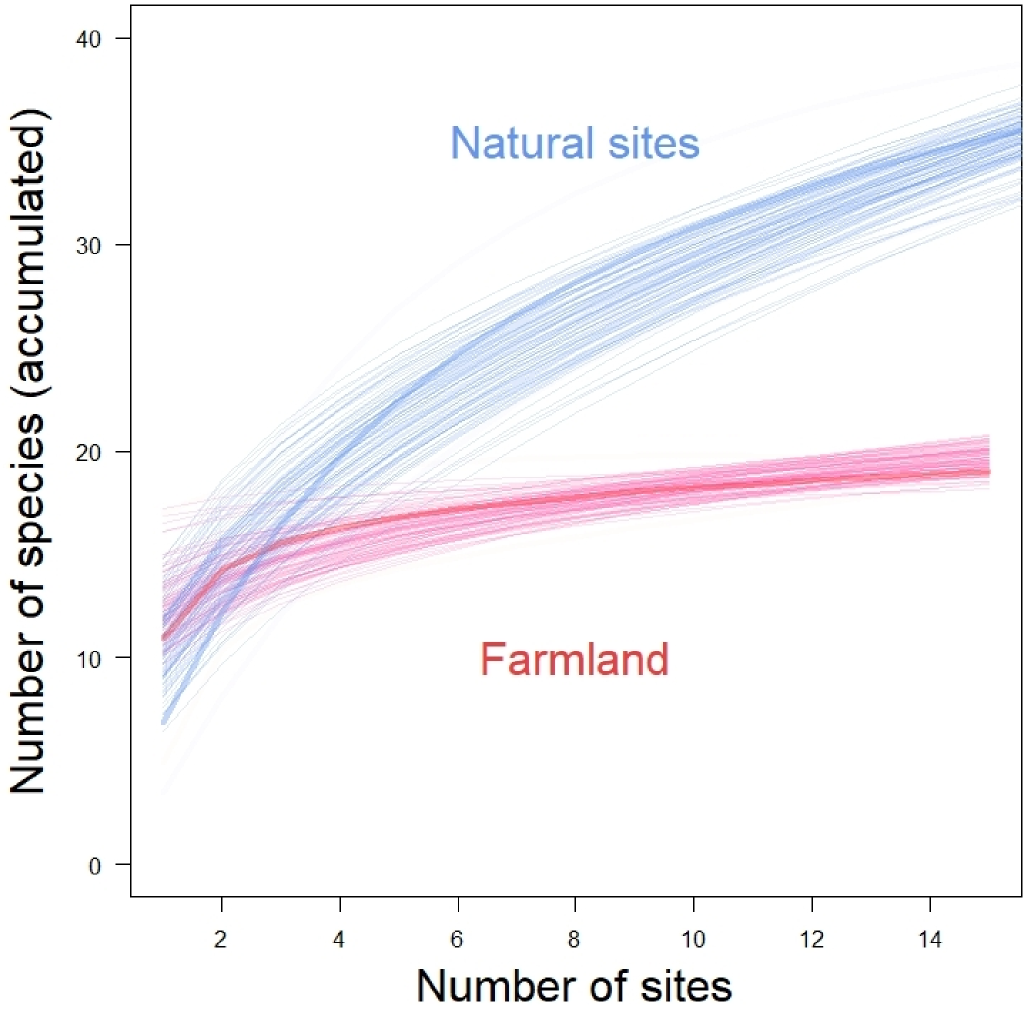

Figure S1. Species-accumulation curves for sites sampled in natural habitats and farmland across Rwanda. The number of species found in farmland approaches a maximum after 15 sites, whereas the number of species found in natural habitats still increases steeply.

Table S1. Functional traits of frog species used in the study. The selection of traits follows Ernst et al. (2006) and Cadotte et al. (2011).

Trait	Scale	Characteristic attributes / Unit
Basic microhabitat of postmetamorph	nominal	1: (semi)aquatic; 2: water edge (pond, river), on ground; 3: water edge/swamp on vegetation <2 m; 4: terrestrial incl. veg ≤ 1 m; 5: arboreal incl. tree holes; 6: semiarboreal; 7: fossorial
Calling site	nominal	1: (semi)aquatic; 2: water edge (pond, river), on ground; 3: water edge/swamp on vegetation <2 m; 4: terrestrial incl. veg ≤ 1 m; 5: arboreal incl. tree holes; 6: semiarboreal; 7: fossorial
Egg deposition site	nominal	1: lentic; 2: lotic; 3: leaf litter; 4: phytotelm/rock pool; 5: burrows/underground; 6: foam nest
Egg deposition type	nominal	1: into water; 2: above water surface; 3: independent of water
Breeding seasonality	ordinal	1: no marked seasonality/throughout year; 2: seasonal (at beginning of rainy season); 3: explosive (for a few days only)
Tadpole	nominal	1: none; 2: free-swimming
Mean snout-vent length male	numeric	Mean [mm]
Mean head width male	numeric	Mean [mm]
Mean snout-vent length female	numeric	Mean [mm]
Mean head width female	numeric	Mean [mm]
Relative hind limb length	numeric	Ratio tibiofibula length/SVL
Hand webbing	ordinal	1: absent or traces; 2: less than half-webbed or not between all fingers; 3: up to two-thirds webbed between all fingers; 4: nearly fully or fully webbed
Foot webbing	ordinal	1: absent or traces; 2: half-webbed; 3: two-thirds-nearly fully; 4: fully webbed
Terminal disks	ordinal	1: not expanded; 2: slightly expanded; 3: expanded

Cadotte M. W., Carscadden K. & Mirotchnick N. (2011) Beyond species: functional diversity and the maintenance of ecological processes and services. – Journal of Applied Ecology 48: 1079–1087.

Ernst R., Linsenmair K. E. & Rödel M.-O. (2006) Diversity erosion beyond the species level: dramatic loss of functional diversity after selective logging in two tropical amphibian communities. – Biological Conservation 133: 143–155.

Supplementary Information 2

Analyses without high-elevation sites (> 2500 m).

Results

Comparison of alpha and gamma diversity

We recorded a total of 42 amphibian species at the study sites: 22 in forest, 17 in savannah and 19 species in farmland. Fifteen species (eleven savannah and four forest species) were shared between natural sites and farmland; three species (*Hyperolius lateralis*, *Ptychadena porosissima*, *P. uzungwensis*) were exclusively found in farmland. Alpha diversity in farmland was higher than in natural sites (*species richness*, farmland: median (interquartile range): 11 (3.5), range 6–17, natural sites: 7 (2.5), 4–10, Fig. S2.1a; *functional diversity*, farmland: 2.24 (1.63), 0.14–3.06, natural sites: 1.26 (2.70), 0.06–7.72, Fig. S2.1b; *phylogenetic diversity*, farmland: 1248 (411), 747–1604, natural sites: 799 (284), 561–1228, Fig. S2.1c). In contrast, gamma diversity was higher in natural sites than in farmland (*species richness*, natural sites 37 (range 35–39), farmland 19, Fig. S2.1a; *functional diversity*, natural sites 20.8 ± 0.73, farmland 3.9, Fig. S2.1b; *phylogenetic diversity* natural sites 2520 ± 57, farmland 1731, Fig. S2.1c).

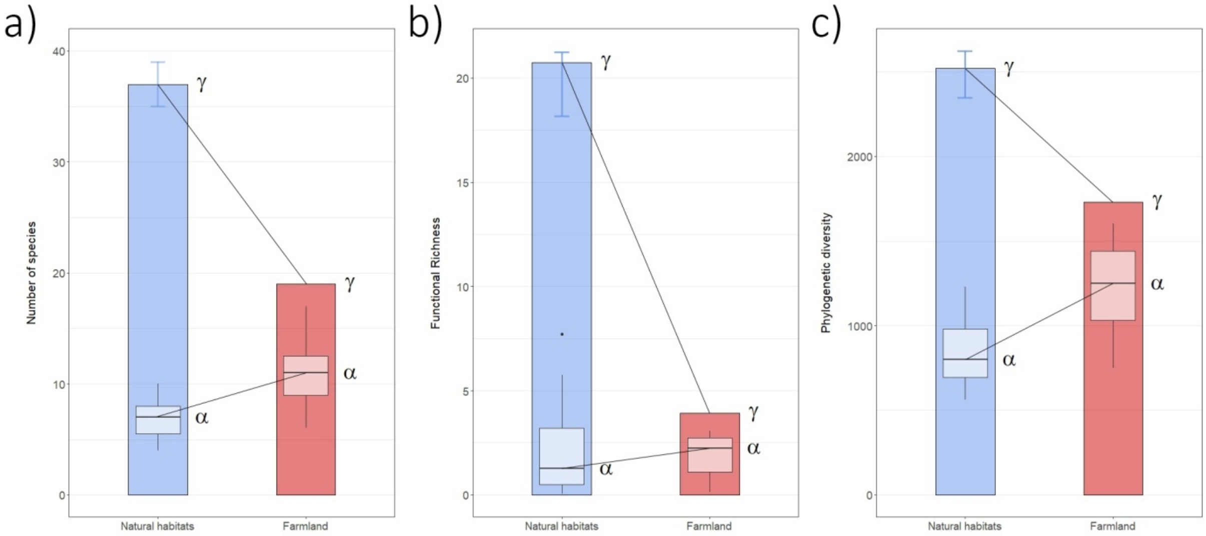

Figure S2. Alpha and gamma diversity of amphibian communities in natural habitats (natural forest, natural savannah; blue) vs farmland (red) across Rwanda. Diversity is measured as (a) taxonomic diversity (number of species), (b) functional diversity (functional richness), and (c) phylogenetic diversity (Faith's PD) both on the level of local communities (alpha diversity) and once pooled across Rwanda (gamma diversity). Gamma diversity is shown as colored bars; median and standard deviation of local alpha diversity (n = 15) are shown as lighter boxplots within bars. Gamma diversity in natural habitats represents the median of 1000 sampled combinations of seven and eight forest and savannah sites; error bars indicate observed range. Alpha diversity was consistently higher in farmland than in the natural habitat, whereas gamma diversity was consistently higher in natural sites than in farmla
